# A Novel Physical Sensing Principle for Liquid Characterization Using Paper-Based Hygro-Mechanical Systems (PB-HMS)

**DOI:** 10.3390/s17071667

**Published:** 2017-07-20

**Authors:** Angel Perez-Cruz, Ion Stiharu, Aurelio Dominguez-Gonzalez

**Affiliations:** 1 Department of Mechanical and Industrial Engineering, Concordia University, 1455 De Maisonneuve Blvd, W. Montreal, QC H3G 1M8, Canada; istih@encs.concordia.ca; 2 Facultad de Ingeniería, Universidad Autónoma de Querétaro, Cerro de las Campanas s/n Querétaro, Querétaro 76000, Mexico; auredgz@uaq.mx

**Keywords:** paper-based sensors, sensing principle, hygro-mechanical system, cantilever beam, hygroexpansive strain

## Abstract

In recent years paper-based microfluidic systems have emerged as versatile tools for developing sensors in different areas. In this work; we report a novel physical sensing principle for the characterization of liquids using a paper-based hygro-mechanical system (PB-HMS). The PB-HMS is formed by the interaction of liquid droplets and paper-based mini-structures such as cantilever beams. The proposed principle takes advantage of the hygroscopic properties of paper to produce hygro-mechanical motion. The dynamic response of the PB-HMS reveals information about the tested liquid that can be applied to characterize certain properties of liquids. A suggested method to characterize liquids by means of the proposed principle is introduced. The experimental results show the feasibility of such a method. It is expected that the proposed principle may be applied to sense properties of liquids in different applications where both disposability and portability are of extreme importance.

## 1. Introduction

Nowadays, different areas demand reliable sensors, which have to perform quick and accurate tasks. One of the main concerns in biological and food quality applications is the detection and/or characterization of liquids (e.g., sugar or alcohol content in beverages and bioassays). The development of sensing elements that must be extremely portable to perform tests at locations where laboratories are not accessible is of extreme importance. In addition, in some applications the sensing elements are usually in contact with the analyte which may be chemically active compounds or hazardous biofluids; therefore, they must be disposed carefully to avoid any biohazards. In this regard, a huge challenge for scientists is to improve or build new techniques for characterizing liquid that must fulfill the aforementioned needs at a low-cost without compromising the performance.

Several physical principles have been applied to characterize liquids. Among the most important in the market are the optical principles including refractometers and spectrometers. The former can be found as ultraviolet-visible (UV-VIS) and near infrared (NIR) spectrometers. These systems use the absorption and/or reflectance over a specific range of wavelength of light to generate a response spectrum that can be used to characterize and identify the components in a liquid. Although some portable devices are available on the market, there are relatively expensive. Another optical principle used to characterize liquids is the measurement of the refraction index. The devices that apply this principle are known as refractometers. Compared to spectrometers, the cost of refractometers is significantly lower. However, refractometers are limited to characterize binary solutions with known components (e.g., water-sugar and water-alcohol solutions). In addition, some other physical principles to perform liquid characterization have been reported in the literature. For instance, the hydrodynamic forces of a vibrating device immersed in a fluid have been used to approximate the density of liquids [1]. In this sensing principle, the reduction of the resonance frequency due to the hydrodynamic forces is measured to estimate the density of the fluid. In another example, the electric properties of liquids have also been applied to perform characterization of vegetal [2] and automotive [3] oils. However, it is expected that many other physical principles could be applied to achieve the characterization and or identification of liquids.

In this work, a novel physical sensing principle to perform characterization of liquids is proposed. The principle takes advantage of the hygroscopic properties of paper to characterize polar liquids. The proposed sensing principle makes use of the particular hygro-mechanical bending response as a result of the interaction between a paper-based cantilever beam and a liquid droplet, named as a paper-based hygro-mechanical system (PB-HMS). It is proposed that the characterization of liquids could be conducted by analyzing the dynamic response of the PB-HMS. In this regard, the dynamic response PB-HMS is modeled through the identification of a high order linear system. Furthermore, a method to apply the proposed sensing principle in order to characterize liquids is proposed. In order to study the feasibility of the proposed PB-HMS to characterize liquids, the study of water-alcohol and water-sugar solutions is discussed. The results of the proposed sensing principle are compared to those achieved using conventional refractometers.

## 2. Background of Paper-Based Systems

In the past decade, serval paper-based sensors and actuators have emerged as an alternative to perform the same tasks microsystems do. The concept of electro-active paper actuators (EAPad) was introduced by Kim et al. [4] in 2000. This system consists of a piece of paper sandwiched between two compliant electrodes. In 2005, Lee [5] presented the first urine-activated paper-based battery. In his work, Lee focused on the need to develop simple devices that may produce energy without complex fabrication processes. Later, Whitesides and co-workers introduced the concept of paper-based microfluidic systems in 2007 [6]. They presented a device to perform multiple analytical assays in a single piece of paper by selectively patterning of channels. Thus, from a general point of view all of these devices may be considered paper-based systems.

Other paper-based systems that used paper as a substrate can also be found in the literature. Liu et al. [7] presented a force sensor fabricated using paper as a structural material. They fabricated carbon resistors on cantilever beams made of chromatography paper. Although they named their device a MEMS sensor, its size (~cm) is quite large compared to the ones made from Si which are micro-scale. An extension of this work was presented by Ren et al. [8]. They showed the feasibility of paper-based sensors fabricated using conventional office paper. Paper-based magnetic actuators by made by impregnating paper with a ferrofluid have been presented by Li et al. [9] and Ding et al. [10]. In another work, Fraiwan et al. [11] developed a paper-based cantilever sensor for volatile organic compound detection. The cantilever beam was fabricated by spin coating a sensitive polymer on the paper. In addition, paper-based sensors using printable electronics have been reported in past years. Mraović [12] developed a capacitive humidity sensor by screen printing conductive electrodes on paper and cardboard. All reported paper-based mechanical systems applied either conventional sensing or actuation principles.

Paper-based systems exhibit several advantages with respect to their micro-system counterparts made from silicon and polymers: (i) paper is inexpensive and most importantly, it is readily available in several types; (ii) the fabrication processes of these devices are simple. In fact, they involve conventional technologies that have been already applied to paper products (i.e., screen, inkjet and laser printing); (iii) as the size of these devices is small, on the scale of millimeters, they are extremely portable; (iv) the volume of sample that is required to perform an analytical assay using PB devices is smaller than that needed when using instruments such as spectrometers and refractometers. The volume is usually in the microliter range for PB devices compared to the milliliter range for the aforesaid optical instruments; (v) microfluidic devices are self-driven by capillary action, and no external hardware is needed to generate a flow; finally, (vi) paper-based systems are readily disposable. Any biological waste can be simply disposed of with a reduced environmental footprint by simple incineration of the device.

From a physical point of view, paper offers unique features that facilitate the development of paper-based systems. Paper-based microfluidic systems have taken advantage of its capability to transport liquids as well as its chemical compatibility to launch several applications [13]. Paper-based mechanical systems have capitalized the flexural properties of paper to generate or to detect motion. However, paper has other physical properties that can also be applied to extend the functionality of paper-based systems. One characteristic of paper that has not been fully explored is the hygroscopicity. In nature, several actuation systems of plants are based on the differential swelling on their walls due to the daily change of humidity in the surrounding environment, known as hygroscopic motion [14,15,16]. These movements include bending, twisting and coiling [14]. Some studies have been executed to study hygroscopic motion in pine cones [17], Geraniaceae plants [18], and *Salaginella lepidophylla* [19]. Since paper is made of natural highly hygroscopic fibers (i.e., cellulose), it is an excellent alternative for developing paper-based hygro-mechanical devices.

In addition, some results of studies regarding the curling response of paper-plastic bilayer actuators activated due to changes in the relative humidity have been reported [17]. Recently, the curling response of surface treated paper due to its interaction with water has been reported [20,21]. The main difference of these works is in the moisture source. Here, the motion is produced by the interaction of water and paper rather than the change of the relative humidity in the environment. In these works a piece tracing paper was deposited on a water bath; then, a virtual bilayer (wet/dry) system is formed as the water rise along its thickness. These works have shown the feasibility to produce motion of paper using water through the hygro-mechanical response. However, these works only focused on the physical study of the phenomenon and the potential applications using this phenomenon have not been discussed.

## 3. Physical Sensing Principle

In this section, the bending response of the PB-HMS is described to provide a context of the proposed physical sensing principle. Then, a detail explanation of the design of the proposed PB-HMS is presented.

### 3.1. Hygro-Mechanical Bending Response

As previously reported in [20,21] the interaction of a piece of tracing paper with water, which is its natural solvent, induces a transient bending response. This response truly depends on the water infiltration into the paper. Two phenomena that are inherent to the water transport determine the shape of the bending state. First, the fibers of paper swell under the influence of water [22]. This phenomenon is found in some plants and is known as hygroexpansive actuation. It has been suggested that swelling takes place as the water molecules break and replace the inter-chain bonds in cellulose [23]. Second, an apparent relaxation of the Young modulus of paper is produced as the moisture content in the fibers rises [24]. This effect is due to the interaction of cellulose chains and water, which acts as a plasticizer that reduces the Young’s modulus [25]. Thus, a combination of these two phenomena yields a specific transient bending response of paper.

The authors in [20,21] focused on the theoretical study of the hygro-mechanical response of tracing paper due to its interaction with water. They provided an explanation of the phenomenon from a physical point of view. However, it is suggested that this phenomenon may be applied to characterize liquids that are of extreme importance in different areas such as food industry; for instance, the determination of alcohol or sugar in commercial beverages. In addition, this phenomenon may be applied to characterize water based biofluids (e.g., urine, saliva, and blood).

### 3.2. PB-HMS Design

**Figure 1:**
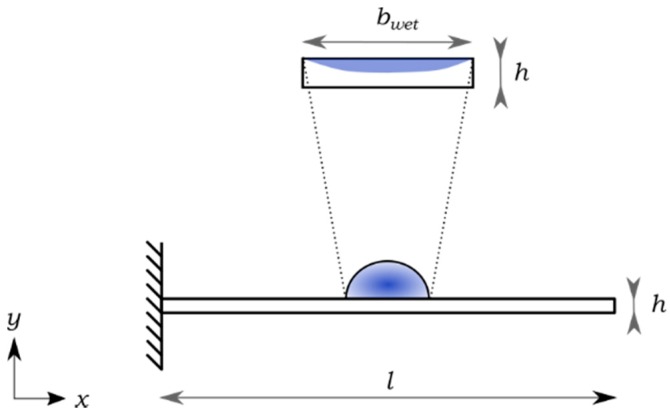
Proposed experimental configuration of the PB-HMS.

The PB-HMS is formed by two elements: a paper-based beam and a water-based liquid. In this regard, the input of the system is the liquid and the output is the bending response. From a practical point of view, the beam-liquid interaction system studied in [20,21], positioning the paper-based beam on a water reservoir, represents a major drawback as the measurement of the bending response requires sophisticated techniques due to the lack of alignment between the beam and the optical detector. In this regard, a local modification of the moisture that selectively modifies the liquid content in paper is proposed. Here, a small droplet of liquid is deposited on the surface of the beam to form the PB-HMS as shown in Figure 1. Here, the active element is a paper-based cantilever beam of length *l* and thickness *h*. The bending response is triggered by a small droplet that wets a portion (*b*_wet_) of the cantilever length. This type of moisture source provides an easy way to modify the moisture content by using a standard mechanical pipette. The volume of the sample is defined as low as 1.5μL such that the evaporation rate is significantly slower compared to the bending response.

**Figure 2:**
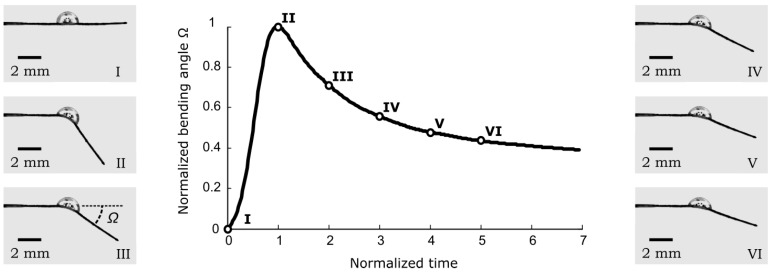
Hygro-bending response (bending angle Ω) of paper-based cantilever beam along its CD due to its interaction with one droplet of water. Time is normalized with respect to the time at which the maximum bending angle occurs (please refer to supplementary material V1).

In order to illustrate the PB-HMS bending response let us consider one system using a 1.5 μL droplet of distilled water (Figure 2) and a tracing paper cantilever beam. The cantilever beam is oriented along its cross direction (CD). It was previously found that the tracing paper on water swells up to 10% in CD, while only it does only 1% in the machine direction (MD) [20,21]. After the droplet is deposited on the cantilever surface, the CD cantilever beam starts to swell non-homogeneously along the thickness producing a bending angle Ω with respect to the horizontal. Then, a maximum curvature is reached. Although the droplet evaporates, the cantilever beam can achieve a steady state curvature.

This kind of response can be described as a single oscillation that may reveal information about the liquid used to trigger the hygro-mechanical motion. Thus, as in the case of the hydrodynamic sensing principle, the proposed principle is based on the sensitivity of the liquid used in the dynamic response of the PB-HMS.

## 4. Method

In order to apply the aforesaid physical sensing principle to characterize liquids a proposed method is described in this section. The main steps are summarized as follows:

(1) Fabricate the sensing element of the PB-HMS.
(2) Measure the PB-HMS dynamic bending angle.
(3) Model the PB-HMS dynamic response due to a particular liquid as a high order linear system by using identification algorithms.
(4) Extract the resonance frequency, gain margin and crossover frequency of the identified model.

### 4.1. Fabrication of the Sensing Element

In order to study the dynamic response of the PB-HMS, two cantilever paper-based beams are fabricated (C1 and C2). The cantilever beam C1 is made of tracing paper (Carson^®^ as used in [20,21]) and the cantilever C2 is made of standard pad paper supplied by a local store. The cantilever beams C1 are made using tracing paper as received, while the beams C2 are filled with an acrylic resin from a permanent marker. This treatment is performed to mimic the structure of tracing paper. All the experiments were carried out in a chamber at a room temperature at 24 °C with a relative humidity of 50%. A set of cantilever beams (length = 9 mm, width = 0.9 mm) were fabricated. The fabrication procedure is extremely simple as it involves only one and two steps for the case of cantilevers C1 and C2, respectively. The main step in both cases is the cutting process. This process was performed using a commercial electronic paper cutter (Silhouette Portrait^®^, Silhouette America, Inc., Lehi, UT, USA). This step process requires less than 5 min to fabricate more than 100 cantilever beams.

**Figure 3:**
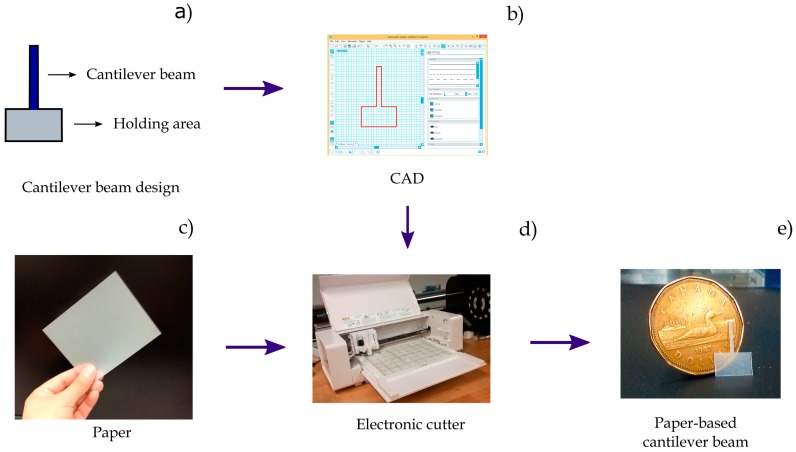
The fabrication process of the paper-based cantilever beams. (**a**) A rectangular area is added to the cantilever beam for holding purposes; (**b**) The design is implemented using CAD; (**c**) The paper is placed on an electronic cutter; (**d**) The paper-based elements are cut; (**e**) A fabricated paper-based cantilever beam is compared to the size of a one Canadian dollar coin.

Figure 3 presents the fabrication process of a cantilever beam made of tracing paper. In the case of cantilever C2, the pad paper is previously filled with acrylic resin using a permanent marker ink.

### 4.2. Measurement of the PB-HMS Dynamic Response

**Figure 4:**
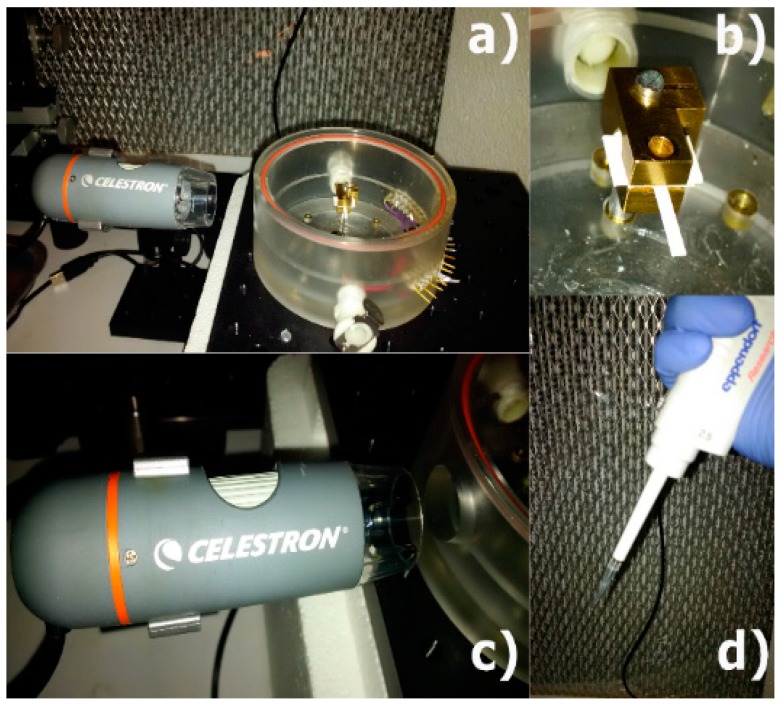
Measurement of the hygro-mechanical bending response. (**a**) Experimental setup and (**b**) a detail of the cantilever holder; (**c**) the portable microscope and (**d**) the mechanical pipette used to deposit the fluid droplet on the cantilever beam surface.

The experimental setup for the measurements carried out is presented in Figure 4. The paper-based cantilever beam is placed in a metallic holder as illustrated in Figure 4b. Then, a digital microscope (Celestron LLC, Torrance, CA, USA is positioned to observe the paper microbeam from the profile and record the out-of-plane motion of the cantilever beam (Figure 4c). Finally, the water droplet is deposited on the cantilever surface at half of the length by using a metered pipette (Figure 4d). In order to extract the hygro-mechanical dynamic response of the system (droplet-cantilever), a sequence of images was acquired using the digital microscope. The sampling recording rate selected for the case of C1 and C2 were 2 and 1 fps, respectively, with a resolution of 2048 × 1536 pixels.

### 4.3. Modelling of the PB-HMS Dynamic Response

Looking at the bending response of PB-HMS presented in Figure 2 it is not hard to determine that such response is very similar to some of the well-known dynamic systems (e.g., electromechanical systems). However, the response of the PB-HMS (beam-droplet) exhibits a very low frequency. Hence, it is proposed that the bending response of PB-HMS can be represented as an equivalent linear system. Let us assume that the response ξ (bending angle, velocity or acceleration) can be described by means of an ordinary linear differential equation of *n^th^* order under a step input (*u*_0_) as described in Equation (1):

(1a) $$A_{n}\frac{d^{n}\xi }{dt^{n}}+\ldots +A_{2}\frac{d^{2}\xi }{dt^{2}}+A_{1}\frac{d\xi }{dt}+A_{0}=B_{n}\frac{d^{n}u_{0}}{dt^{n}}+\ldots +B_{2}\frac{d^{2}u_{0}}{dt^{2}}+B_{1}\frac{du_{0}}{dt}+B_{0}$$

(1b) $$u_{0}\left(t\right)=\{\begin{array}{cc} 0 & t<0\\ 1 & t\geq 0 \end{array}$$

The coefficients of Equation (1) can be easily obtained using experimental data and applying optimization algorithms. The approximation of such coefficients in this work is performed by means of the identification toolbox of MatLab^®^ (The Mathworks Inc., Natick, MA, USA). The accuracy of the approximation in this toolbox is measured by the “fit,” which is the normalized root mean square error and is defined as:

(2) $$fit=\left[1-\frac{\sum \left(Y-Y_{p}\right)}{\sum \left(Y-\bar{Y}\right)}\right]\times 100$$

where *Y* represents the experimental amplitude of the bending acceleration, $Y_{p}$ stands for the value of the output of the transfer function, and $\bar{Y}$ denotes the mean value of the measured signal. In the case of $\sum \left(Y-Y_{p}\right)=0$ a perfect match of 100% is found.

### 4.4. Evaluation of the PB-HMS Dynamic Performance

The most common way to investigate the dynamic response of the hygro-mechanical system is to analyze the frequency response. The hygro-mechanical system exhibits a very low frequency response (mHz) which is almost six orders of magnitude lower that of typical microcantilever sensors using the hydrodynamic principle (kHz). Perhaps the simplest parameter to characterize liquids is the resonance frequency (*fr*) of the hygro-mechanical system. For instance, an alternative approach to characterize the binary aqueous solutions is to evaluate the shift of *fr* as the concentration of the solution is modified. Such a method has been applied to characterize the performance of microcantilever beam sensors [1].

**Figure 5:**
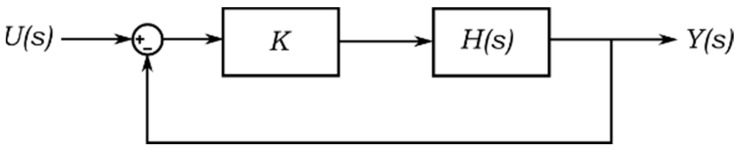
Close loop configuration to study the virtual stability.

Furthermore, the stability that describes the performance of a system may also be applied to quantify the response of the system. The hygro-mechanical motion produces a stable response of the PB-HMS as depicted in Figure 2. However, it is possible to evaluate the virtual response of the identified PB-HMS to evaluate its stability. In other words, one may evaluate several virtual bending experiments under different hypothetical scenarios to study the stability. One of these possible scenarios is the close-loop configuration of the hygro-mechanical system, which is usually applied to control similar systems, see Figure 5. The stability study can be performed by means of the parameters extracted from the frequency spectrum such as the gain margin (Gm). According to Nagurka and Kurfess [26], the gain margin is defined as “the number of decibels by which the magnitude of the open loop frequency response falls short of unity when the phase angle is 180”. The frequency where the phase angle is 180 degrees is often known as crossover frequency. Thus, in addition to the resonance frequency, the gain margin and its crossover could be applied to quantify the performance of the PB-HMS.

## 5. Results and Discussion

### 5.1. Dynamic Performance of the PB-HMS

**Figure 6:**
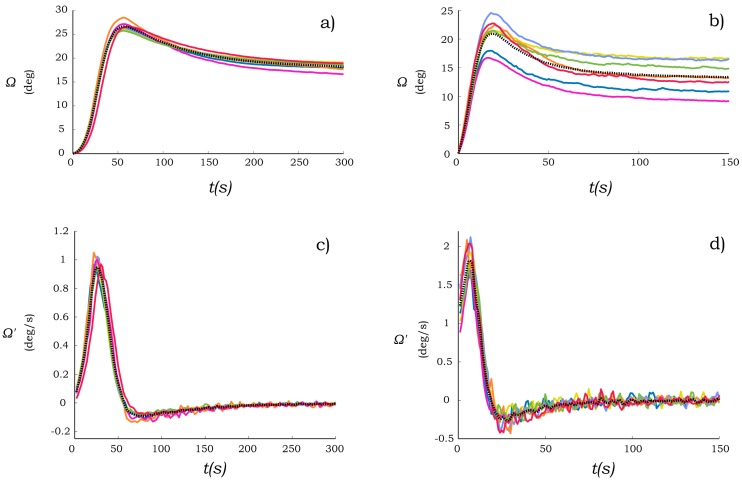
Hygro-bending response of paper-based cantilever beam. Bending angle of (**a**) C1 and (**b**) C2. The first derivative with respect to time of the bending angle of (**c**) C1 and (**d**) C2. Line colors represent different experiments (*N* = 7) and dotted line the average.

The measured bending angle response of the PB-HMS using cantilevers C1 and C2 due to their interaction with a 1.5 μL droplet of distilled water is presented in Figure 6a,b, respectively. The characteristic bending response of both cantilever beams is similar to that found in [20] for the case of paper-based beams placed on the surface of a water bath. However, it can be seen that the steady state bending angle does not come back to the initial straight configuration as reported in [20]. This effect may be due to the fact that the mass of both beam and droplet became significant for the proposed cantilever beam configuration. These loads would play a major role in the bending response as soon as the paper softens due to the water penetration in the paper. Both cantilever beams yield a similar bending angle response, suggesting that this phenomenon is not an exclusive property of tracing paper.

It can be noted in Figure 6a,b that the bending angle response of the cantilever C1 has a slight deviation when performing several experiments (*N* = 7). In contrast, the response of the cantilever C2 has a significant deviation. Such effect is reasonable due to the fact that the cantilever C2 is made of pad paper that has a less consistent quality than the tracing paper. In addition, the result may be influenced by the filling process. Although this deviation in the bending angle has been observed, it can be noted that the characteristic shape of the response appears to be unaffected. This effect suggested that such a deviation is dominated by the maximum hygroexpansive strain actuation, which only modifies the amplitude of the response without affecting the shape of the bending state.

To further study the dynamic response of the paper-based cantilever beams the first derivative of the bending angle with respect to the time, namely bending velocity, is presented in Figure 6c,d. These velocities were estimated from the bending angles measurements using the centered differentiation numerical method. It can be noted that such bending velocities can be described as a single oscillation of a dynamic system in the range of mHz. Such a frequency is very low; it is two order of magnitude lower compared to typical mechanical systems such as bounce of ground vehicles or in the heart beating in one adult. It can be seen in Figure 6d that in the bending velocity the significant deviation presented in the bending angle is compensated. Thus, it is suggested that to avoid deviations due to a variation on the hygroexpansive strain actuation; the bending velocity would represent a better choice to characterize the dynamic response of the paper-based beams.

### 5.2. Liquid Sensitivity

**Figure 7:**
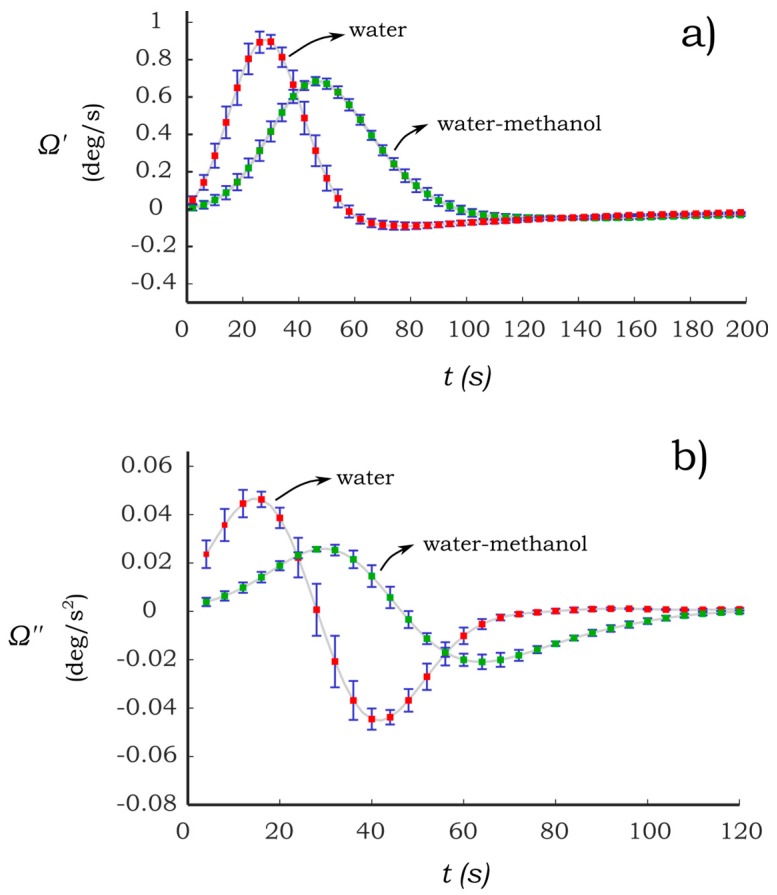
Dynamic response of paper-based cantilever beam C1 under the influence of water and water-methanol (**a**) bending velocity (Ω′); and (**b**) bending acceleration (Ω″). Bars represent the standard deviation for *N* = 7.

**Figure 8:**
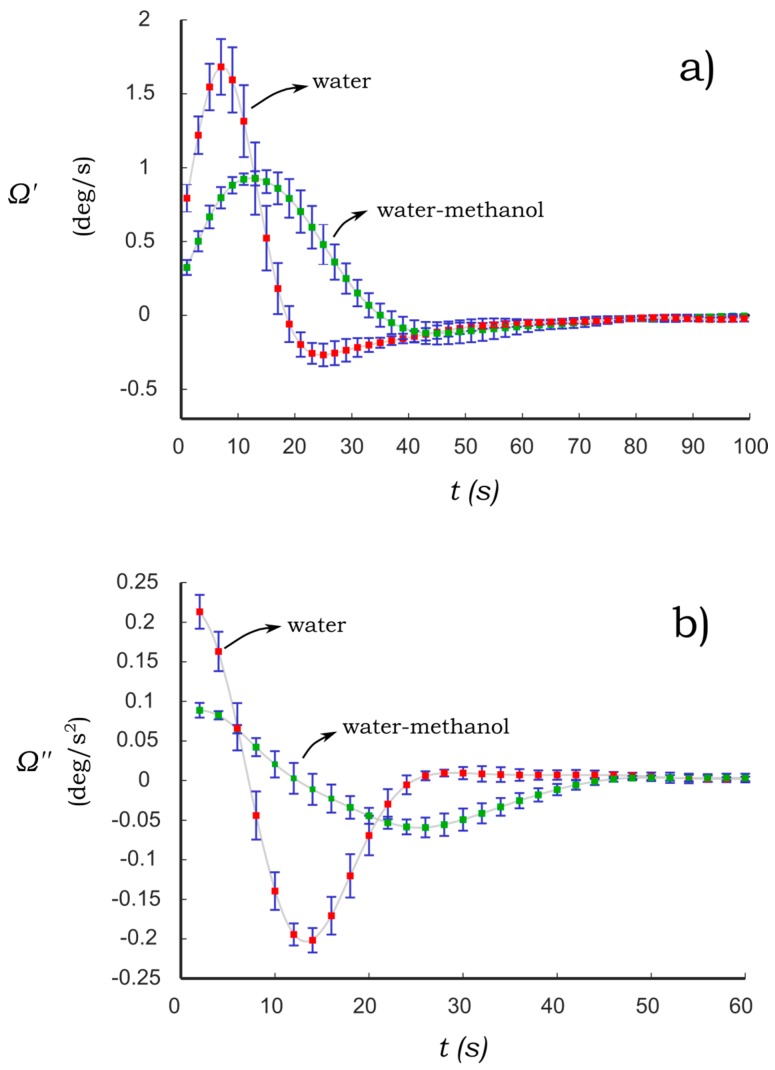
Dynamic response of paper-based cantilever beam C2 under the influence of water and water-methanol (**a**) bending velocity (Ω′); and (**b**) bending acceleration (Ω″). Bars represent the standard deviation for *N* = 7.

Another important aspect to be considered is the sensitivity of the paper-based cantilever beam to modifications in the concentration of binary solutions. The feasibility of the proposed sensing principle is based on the sensitivity of the PB-HMS to changes in the concentration of the wetting solutions. In this regard, the dynamic response of cantilevers C1 and C2 due to their interaction with distilled water and water-methanol are presented in Figure 7 and Figure 8. The former binary solution was prepared at a concentration of 20% (*v*/*v*). The second derivative of the bending angle is also presented in Figure 7 and Figure 8 as they provide an extended means to evaluate the dynamic response.

In Figure 7, it can be seen that C1 is sensitive to the presence of methanol at the given concentration. The analysis of the response of the binary solution can be performed by taking as reference the response of pure water, see Figure 7a. The dynamic response of the methanol solution appears to experience a delay in the time response. This effect is more evident if one looks at the bending acceleration in Figure 7b. Here it can be seen that the time duration of the bending acceleration is higher compared to that of the water response. In addition, a shift in time of the maximum and minimum bending acceleration is induced by the addition of methanol as can be seen in Figure 7b. It is also remarkable that the amplitude of the acceleration is significantly reduced compared to the case of the pure water. In this case, these effects may be due to the properties of the liquid such as viscosity and surface tension.

The dynamic response of the cantilever C2 due to its interaction with water and water-methanol is presented in Figure 8. It can be noted that the bending acceleration (Figure 8b) produced by water is similar to that corresponding to C1 (Figure 7b). Hence, it is suggested that the filling process of C2 has not a significant influence on the characteristic bending response of the PB-HMS using water. By adding methanol, the bending response (Figure 8) is modified in both time response and amplitude. This effect is notable in the bending acceleration as can be seen in Figure 8b. As in the case of C1, the peaks of maximum and minimum acceleration are reduced almost to half of those corresponding to water. This implies that the three hygro-mechanical response of water-methanol has similar behavior for both types of cantilever beams C1 and C2.

### 5.3. Characterization of Binary Aqueous Solutions

In order to show the feasibility of the proposed method to characterize liquids, two sets of binary aqueous solutions are analyzed using the method described in Section 3. The first set solution is made of methanol at 10, 20, 30, and 40% (*v*/*v*). The second set was prepared using sucrose at 30, 60, 90, 120, and 150 g/L. Cantilever C2 was chosen to perform characterization of liquids as it provides a faster response compared to cantilever C1 as described in the previous section. The time response of the bending acceleration response of C2 is almost half of that corresponding to C1, see Figure 7 and Figure 8.

#### 5.3.1. Identification of the Dynamic Response of the PB-HMS

**Figure 9:**
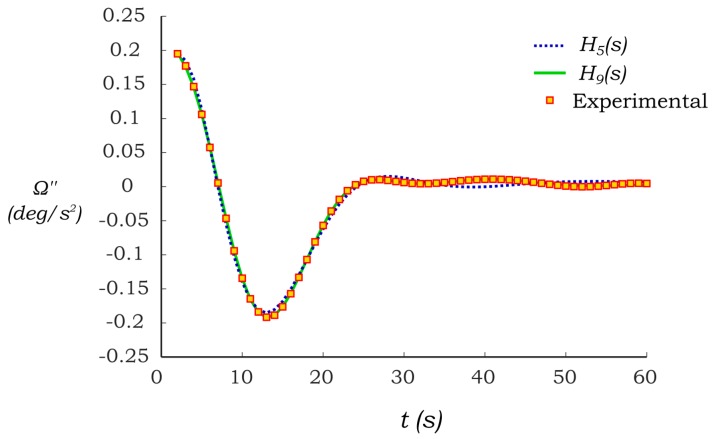
The bending response of the identified PB-HMS.

Table 1 shows different attempts to fit the experimental results of C2 under the influence of water. From Table 1, it can be seen that the two best fits are obtained when using the H_5_(s) and H_9_(s) systems, which fits are 91.69% and 98.83%, respectively. The comparison of both systems responses with respect to the experimental bending acceleration is presented in Figure 9, where it can be seen both identified systems provide good accuracy. H_5_(s) was chosen to perform the identification as it provides a good accuracy in the dominant part of the oscillation as shown in Figure 9. It was found that all the identified systems provide similar accuracy as in the case of water when using H_5_(s).

#### 5.3.2. Evaluation of the Dynamic Performance

**Figure 10:**
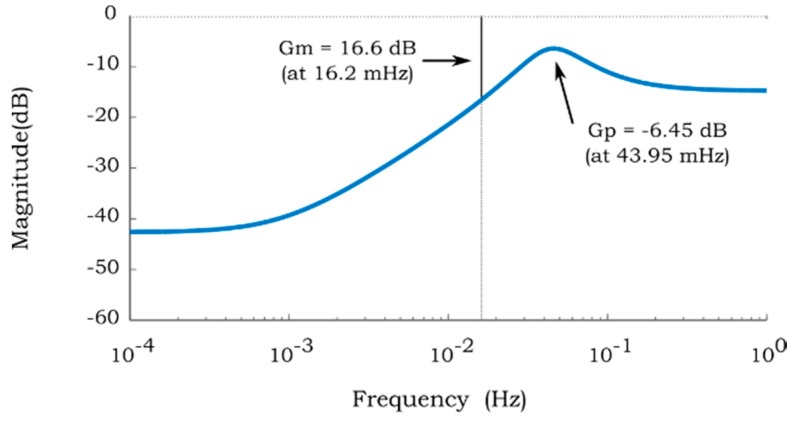
Bode plot of the Identified PB-HMS presented in Figure 8.

The corresponding resonance frequency, gain margin, and crossover frequency of the PB-HMS under discussion are illustrated in the Bode plot presented in Figure 10. These parameters may be taken into consideration to quantify the performance of the hygro-mechanical system. Thus, the stability analysis may be performed to estimate the gain margin and crossover frequency. The gain margins of all the identified hygro-mechanical systems were estimated. It was found that the sensitivity of the close loop hygro-mechanical system becomes more robust as the water content in the binary solution decreases.

Although the stability study does not represent any physical response of the PB-HMS, this behavior is consistent with the fact that the motion of the system is triggered by water. In other words, less water content would yield a more stable response of the PB-HMS.

#### 5.3.3. Characterization Charts

**Figure 11:**
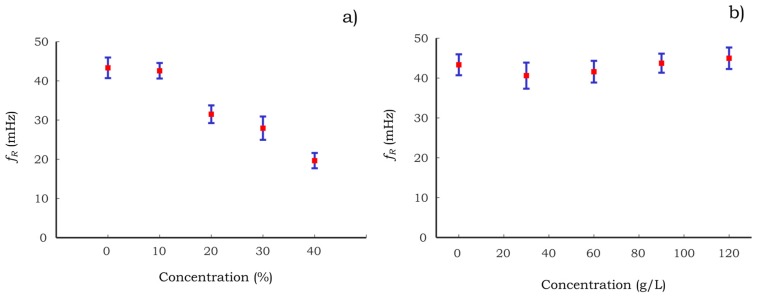
The resonance frequency of the identified systems using C2 (**a**) water-methanol and (**b**) water-sucrose. Red square points represent the average of the experimental measurements and blue lines the standard deviation using *N* = 7.

The correlation between the concentrations of both aqueous solutions with respect to the resonance frequency (*fr*) is presented in Figure 11. The resonance frequency of the systems is obtained analyzing the dynamic response of the identified system in MatLab^®^. For instance, for the case of Figure 10 *fr* is estimated at 43.95 mHz where the maximum gain peak is found. Here, the standard deviation of the resonance frequency is relatively high. This source of deviation may be due to the quality of the paper and/or the filling process of the acrylic resin used to fabricate cantilever C2. It can be seen in Figure 11a that the resonance frequency decreases as the methanol concentration increases. In contrast, the *fr* of the sugar solutions (Figure 11b) exhibits a very low sensitivity to the increment of sucrose content. This effect is associated with the viscosity of the solution that governs the speed of the imbibition of the liquid into the paper. For instance, the water-sucrose solution at 120 g/L has an increment of the viscosity of 8% with respect to pure water. In contrast, the water-methanol solution at 40% has an increment of 81%. Thus, it is suggested that the *fr* of the hygro-mechanical system would not be suitable to estimate the concentration of a binary solution with low viscosity variation as the case of water-sucrose solutions presented in this work.

**Figure 12:**
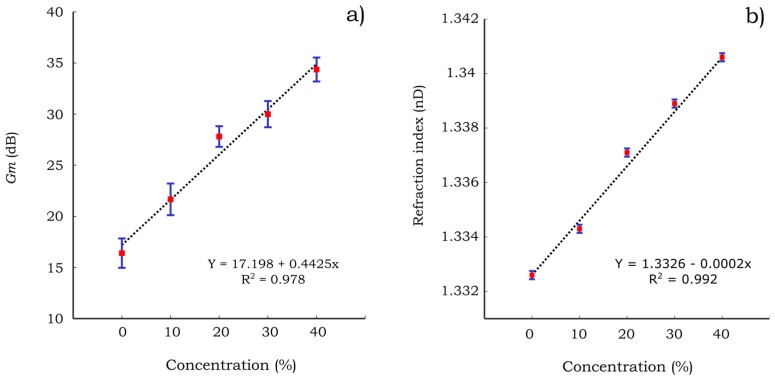
Characterization of water-methanol solutions by means of (**a**) gain margin of PB-HMS (**b**) refraction index. Red square points represent the mean of the experimental measurements and blue lines the standard deviation using *N* = 7.

Moreover, the gain margin of the PB-HMS corresponding to the water-methanol solutions is presented in Figure 12a. The gain margin increases almost linearly with respect to the methanol concentration. It is suggested that the identified hygro-mechanical system becomes more stable (high values of gain margin) as the water content in the binary solution decreases. Although such stability does not represent any physical response of the hygro-mechanical system, this behavior is consistent with the fact that the motion of the system is triggered by water. In other words, less water content would lead to a more stable hygro-mechanical system yielding an increment in the gain margin. A comparison with the characterization of the methanol solutions by means of the refraction index is presented in Figure 12b. The refraction indices were measured using a digital refractometer (DR 301-95®, Kruess GmbH, Hamburg, Germany). This instrument is one of the most common equipment to characterize aqueous solutions by means of an optical principle. The gain margin relationship with the methanol concentration is similar to that corresponding to the refraction index. Hence, it is suggested that this parameter may be applied to characterize the concentration of the water-methanol solutions.

**Figure 13:**
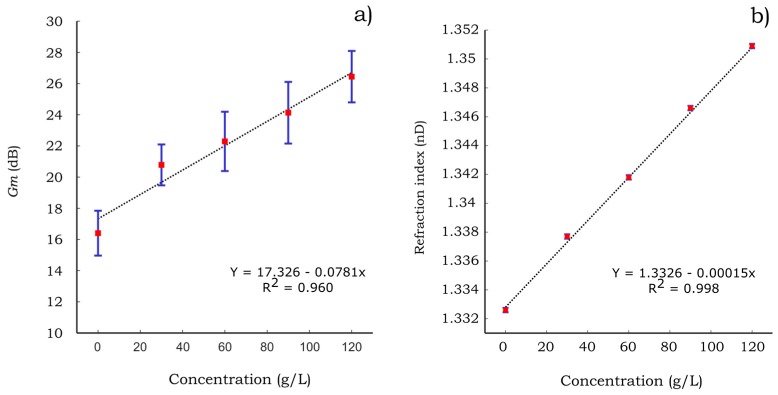
Characterization of water-sucrose solutions by means of (**a**) gain margin of PB-HMS (**b**) refraction index. Red square points represent the mean of the experimental measurements and blue lines the standard deviation using *N* = 7.

Furthermore, the gain margin of the hygro-mechanical systems corresponding to water-sucrose solutions and its comparison with the refraction index characterization are presented in Figure 13. The gain margins of the sugar solutions exhibit a similar trend as the case of methanol solutions when increasing their concentration. However, the standard deviation of the gain margin measurements is higher compared to those obtained for methanol solutions.

The gain margin appears to be sensitive to both aqueous solutions (methanol and sucrose) compared to the resonance frequency that showed good sensitivity to methanol solutions only. As can be seen in Figure 12 and Figure 13 the PB-HMS is more sensitive to the methanol solutions than it is to the sucrose solutions within the studied concentration ranges. For these ranges, the sensitivity of methanol solutions is almost six times higher than that corresponding to the sucrose solutions. In contrast, for the case of the characterization using the refraction index, both solutions exhibit a similar sensitivity for the given range of concentrations. It is expected that the sensitivity to sucrose solutions may be increased by modifying the type of paper and/or the filling polymer. Moreover, it can be seen in Figure 12 and Figure 13 that the deviation of the gain margin (*Gm*) is extremely high compared to that of the index refraction (*nD*). However, considering that the design of the paper-based cantilever beams could be improved, it is expected that this drawback may be minimized. Despite the associated drawbacks on the preliminary results such as high deviation and low sensitivity to sucrose solutions, it is expected that the measurement of the gain margin may be applied to estimate the concentration of binary aqueous solutions.

## 6. Conclusions

A novel physical sensing principle using PB-HMS is introduced in this work. Besides the well-known advantages of paper-based systems (i.e., low cost and ready disposability), one significant advantage of the proposed PB-HMS is the simplicity of the hardware involved in the measurements. The bending response of the paper-based beam is recorded using a portable digital microscope. Other digital cameras including webcams or cell phones could be adopted to further simplify the proposed hardware. It is interesting to remark that the excitation of the sensing element is generated by the interaction of the substrate and the polar liquid. In other words, no external energy (i.e., electrostatic or magnetic fields) is needed to produce the bending response of the PB-HMS.

The applicability of the proposed principle to characterize liquids is discussed in this work by means of the dynamic response of the PB-HMS. The experimental results using two types of paper-based cantilever beams (C1 and C2) suggest that the proposed PB-HMS are sensitive to the water contend in a liquid. Hence, the dynamic bending response of the PB-HMS can be applied to characterize and/or identify binary solutions by means of its dynamic response. In this regard, a method for the characterization of liquid using the dynamic response of the PB-HMS is proposed in this work. By analyzing the virtual response of the gain margin of the system can be evaluated and used to characterize the water content of the binary solution. However, the improvement of the sensitivity and reproducibility of the PB-HMS is suggested in order to develop future applications.

The characterization of liquids by means of the PB-HMS has some advantages against conventional refractometers. First, the sample volume to perform the characterization is remarkably small. The required volume to characterize a liquid using the hygro-mechanical detection is 1.5 μL, i.e., three orders of magnitude smaller than that necessary to measure the refractive index using a digital refractometer. Second, the hygro-mechanical characterization eliminates the possibility of cross contamination as the sensing elements are disposable. These features may be of interest in biological applications, where biohazardous analytes can be examined using extremely small volumes, usually in the range of μL. Finally, it is expected that the proposed PB-HMS may also be applied in other fields that required portable and disposable sensing elements such as food testing and biological assays.

## Figures and Tables

**Table 1:** Fits values of the different identified hygro-mechanical systems.

Transfer Function	Number of Poles	Number of Zeros	Fit
H_1_(s)	2	1	38.15
H_2_(s)	2	2	72.62
H_3_(s)	3	1	73.73
H_4_(s)	3	2	83.16
H_5_(s)	3	3	91.69
H_6_(s)	4	1	87.84
H_7_(s)	4	2	46.29
H_8_(s)	4	3	42.6
H_9_(s)	4	4	98.83

## Supplementary Material

Video of the paper-based system in response to hygro-expansive strain (V1), it is available online at http://www.mdpi.com/1424-8220/17/7/1667/s1.
